# Effect of sintering process on color parameters of nano-sized yttria partially stabilized tetragonal monolithic zirconia

**DOI:** 10.4317/jced.55034

**Published:** 2018-08-01

**Authors:** Niwut Juntavee, Surawut Attashu

**Affiliations:** 1 Department of Prosthodontics, Faculty of Dentistry, Khon Kaen University, Khon Kaen, Thailand; 2 Division of Biomaterials and Prosthodontics Research, Faculty of Dentistry, Khon Kaen University, Khon Kaen, Thailand

## Abstract

**Background:**

Sintering process is responsible for aesthetic of zirconia restoration. This study evaluated the effect of different sintering temperatures and sintered-holding times on color parameters of monolithic zirconia.

**Material and Methods:**

One hundred and thirty five zirconia bar specimens (width-length-thickness = 10×20×1.5 mm) were prepared from yttria-stabilized tetragonal zirconia polycrystalline (Y-TZP) ceramic and randomly divided into nine groups to be sintered at different temperatures [decreasing- (SD, 1350°C), regular- (SR, 1450°C), and increasing- (SI, 1550°C) sintering temperature] and different sintered-holding times [shortening- (HS, 60 min), regular- (HR, 120 min), and prolonged- (HP, 180 min) sintered-holding time]. Color appearance (ΔE), translucency parameter (TP), contrast ratio (CR), and opalescence parameter (OP) were determined with spectrophotometer. An analysis of variance (ANOVA) and Tukey’s multiple comparisons were used to determine for statistically significant difference of color parameters (α=0.05). Crystal sizes were microscopically examined using scanning electron microscope (SEM), and phase composition of zirconia was determined using X-ray diffraction (XRD).

**Results:**

The mean±sd for ΔE, TP, CR, OP were 82.28±1.27, 1.4±0.13, 0.982±0.004, 1.25±0.15 for SDHS, 78.38±0.74, 2.16±0.10, 0.967±0.005, 1.90±0.11 for SDHR, 74.43±0.91, 2.24±0.10, 0.964±0.004, 1.94±0.09 for SDHP, 76.31±1.22, 3.03±0.10, 0.945±0.003, 2,50±0.09 for SRHS, 74.51±1.27, 3.19±0.17, 0.942±0.003, 2.65±0.16 for SRHR, 73.94±0.49, 3.42±0.10, 0.937±0.003, 2,83±0.09 for SRHP, 76.30±0.43, 3.16±0.09, 0.937±0.002, 2.48±0.09 for SIHS 76.73±1.15, 3.05±0.20, 0.939±0.005, 2.38±0.17 for SIHR, and 75.32±1.37, 2.95±0.18, 0.942±0.006, 2.33±0.15 for SIHP. The ΔE, TP, CR, and OP were significantly affected by altering sintering temperatures and holding times (*p*<0.05). Increasing sintering temperature and extending sintering time significantly improved color appearance, translucency, contrast, and opalescence of Y-TZP (*p*<0.05) as evidenced by enlarging grain size and increasing t→m phase shift.

**Conclusions:**

Raising sintering temperature and prolonging sintering time lead to better color appearance, translucency, contrast and opalescence of nano-sized monolithic Y-TZP, and are suggested for sintering process.

** Key words:**Color appearance, contrast, monolithic zirconia, opalescence, sintering process, translucency.

## Introduction

The increasing aesthetic requirements in dental profession have been escalating in the development of new ceramic materials for dentistry. All ceramic restorations have been increasingly using as alternative to metal-ceramic restorations primarily due to their admirable aesthetics, chemical stability, and biocompatibility (1). Anyhow, the brittle nature of ceramic has limited their extensive use in restorative dentistry. Some innovative ceramics, for instance glass-infiltrated alumina, leucite reinforced ceramic, and lithium disilicate glass ceramic have been predictably used for single crown or short span bridge since they do not possess sufficient strength for large restorations. Recently, zirconia based ceramic restoration becomes increasing popular due to its unique reliable strength to use for extensive reconstruction (2). Normally, zirconia consists of polymorphic crystal structure and exists in three forms including cubic (c), tetragonal (t), and monoclinic (m). The m-phase exists at room temperature up to 1170°C. Above that, the crystal structure transforms to t-phase which is morphologically stable until passing 2370 °C where the c-form exists up to its melting point at 2680 °C (3). The t- to m- phase transformation is arisen in pure zirconia during solidification, which results in volume expansion for approximately 3–5%. Nonetheless, zirconia can be controlled to solidify in t-phase at room temperature by adding 3% of Y2O3, leading to an innovative yttria partially stabilized tetragonal zirconia polycrystalline (Y-TZP) ceramic, that is capable of shielding crack propagation through transformation toughening process, which gives rise to having high fracture resistance to withstand masticatory force in extensive reconstruction. The Y-TZP restorations are fabricated from computer-aided design and computer aided manufacturing (CAD-CAM) using either partially sintered block, which requires further sintering to reach fully crystallization or fully sintered block, which do not need further sintering process, but it causes rapid wear of milling machine (4). Soft milling is favor for machining, but may produce less precise restoration due to sintering shrinkage of zirconia (5). The Y-TZP possesses opaque white color appearance with low translucency, thus requires glassy ceramic veneering to achieve a natural appearance (6). Since veneering ceramic usually has low fracture toughness to withstand tensile stress, thus delamination or chipping of veneering ceramic frequently occurs, leading to a frustrating complication in dental practice (7). Monolithic translucent zirconia was introduced in order to eliminate the problem of ceramic delamination, however aesthetic achievement in simulating optical characteristic of natural tooth is still limited. Recently, high translucent monolithic zirconia was generated and gained attracting attention due to their unique optimization of optical and mechanical properties by introducing nano-sized crystalline structure of less than 500 nm to eliminate light scattering effect for providing an optimal aesthetic appearance (8).

The optical behavior of a translucency monolithic Y-TZP needs to be similar to that of the natural tooth in order to achieve aesthetic appearance. Nevertheless upon the fabrication process several factors influence color appearance of Y-TZP such as particle size, heat rate, sintering temperature and sintered holding time. Among them, the sintering temperature and sintered holding time are the prime factors influencing densification and microstructure of nano-sized Y-TZP (9,10). The color appearance primarily depends on the spectral reflectance generated from light scattering at the surface which intensely influences color appearance of ceramic, and can be scientifically quantified by using the Commission Internationale de l’Eclairage (CIE) system since the human perceptibility of color appearance seems to be subjective (11). The quantitative difference of color appearance (∆Ediff) that indicated “clinically imperceptible” as ∆Ediff<3, “clinically acceptable” as ∆Ediff 3-5, and “clinically unacceptable” as ∆Ediff>5 seems practical for clinical practice (12). Color in dentistry also perceives in term of translucency, contrast, and opalescence as fundamental parameters for tooth color selection in clinical practice. Translucency is designated as a relative amount of light transmission or diffuse reflectance from the surface through a translucent material, which relatively associated with light scattering effect as a result of composition, crystalline structure, inclusions, pores, and density of material in relation to the wavelength of the incident light (10,13,14). The ceramic would appear to be opaque, once the majority of light travelling through the ceramic is diligently scattered and dispersedly reflected. On contrary, it would appear to be translucent, if most of the light intensely transmitted through the ceramic, with the minimal amount being scattered or diffuse reflected (15). The Y-TZP is composed of polycrystalline structures that have different refractive indexes and non-homogeneity of crystals, thus it usually exhibits intense scattering and diffuse reflectance, and provokes opacity (15). Translucency is generally determined from the translucency parameter (TP) and contrast ratio (CR) (16,17). The TP indicates the color difference of material at a given uniform thickness upon the black and white background, and relatively corresponds with visual assessment of translucency. Likewise, the CR is the ratio between the reflectance of a specimen on a black background to that on a white background of a known reflectance. The TP=0 indicates an absolutely opaque material, while higher TP value indicates higher translucency of material. The CR=0 means completely transparent material where as CR=1 indicates absolutely opaque material. Opalescence is an optical characteristic of ceramic that manifests as a bluish appearance upon spectral reflectance and orange-brown appearance upon spectral transmittance that generated from scattering effect of wave length of visible light that equally or shorter than the particle size, and is determine by opalescence parameter (OP) (18).

The optical improvement for Y-TZP restoration is feasible for clinician through the sintering process (19). Optical appearance of conventional Y-TZP has been reported for better improving translucency by modifying sintering parameters which directly affects the microstructure and properties of zirconia (20-22). It was described that variation in sintering temperature and sintered-holding time may affect the grain size, microstructure, and possibly affecting the optical properties zirconia (23-25). As the grain size enlarges, zirconia may spontaneously turn into vulnerable t- to m- phase transformations, which may induce alteration in optical appearance (26-28). This is crucial in dental research especially after the introduction of short sintering cycles from the manufacturers. Moreover, the effect of these changes on the optical property of nano-sized monolithic zirconia still remains questionable. This study aimed at investigation whether the alteration in sintering temperature and sintered-holding time of the nano-sized monolithic Y-TZP affect the optical properties. The null hypotheses were that varied sintering temperature and sintered-holding time would not affect the color appearance, translucency, contrast, and opalescence of nano-sized monolithic Y-TZP ceramic.

## Material and Methods

-Preparation of zirconia specimen

One hundred and thirty five (135) zirconia specimens (12 mm width, 25 mm length and 1.8 mm thickness) were prepared into bar shape from partially sintered Y-TZP blanks Pre-shade A3 (VITA YZ HT color®, Vita Zahnfabrik, Säckingen, Germany) using precision machine (Isomet® 1000, Beuhler, Lake Buff, IL, USA), ground down with a silicon carbide abrasive paper until reaching 2400 grit particles, and polished with 1 µm diamond suspension using a polishing machine (Ecomet®3 polisher, Beuhler, Lake Bluff, IL, USA). All specimens were randomly divided into nine groups (15 bars per group) to be sintered according to the combination of three different sintering techniques: decreasing- (SD, 1350°C), regular- (SR, 1450°C), and increasing- (SI, 1550°C) sintering temperature, and three different sintered-holding times: shortening- (HS, 60 minutes), regular- (HR, 120 minutes), and prolonged- (Hp, 180 minutes) sintered-holding time in a furnace (inFire® HTC, Sirona Dental Systems GmbH, Bensheim, Germany) at 17°C/min of heating and cooling rate, to derive for fully sinter bars (10 mm width, 20 mm length and 1.5 mm thickness) due to 20% volumetric shrinkage.

-Determination of color parameters

The specimens were determined for color appearance (∆E), translucency parameter (TP), contrast ratio (CR), and opalescence parameter (OP) at five locations for each specimen using spectrophotometer (ColorQuest® XE, Hunter Associated Laboratory, Reston, VA, USA) with the CIE light illuminant D65, at 6504 K of color temperature, 300-780 nm standard wavelength, 10 degrees observer angle, and 4 mm in diameter of aperture size. A clear plastic jig was used for positioning of each specimen to be exactly directed to the center of the aperture.

-Color appearance (∆E)

**Figure 1:**
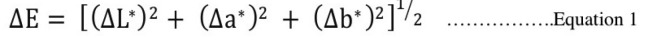
Equation 1.

Color appearance (∆E) was determined from CIE L*a*b* color coordinate difference in lightness (∆L*), red-green (∆a*) and yellow-blue (∆b*), shown in equation 1: (Fig. 1).

-Translucency parameter (TP)

**Figure 2:**
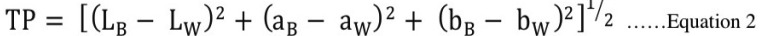
Equation 2.

The CIE L*a*b* color coordinates for each specimen upon standard white [(W), CIE L* = 96.7, a* = 0.1, b* = 0.2] and black [(B), CIE L* = 10.4, a* = 0.4, b* = 0.6] background were used to calculate for TP as equation 2 (16): (Fig. 2).

-Contrast ratio (CR)

**Figure 3:**
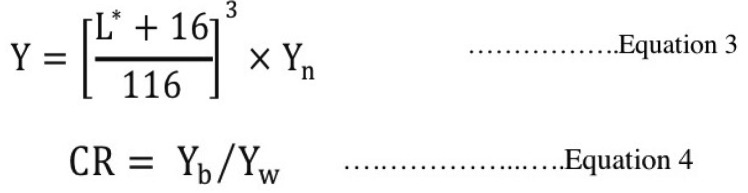
Equations 3,4.

The spectral reflectance [(Y), luminance from Tristimulus color system] was determined from L* values, shown in equation 3 (11,16). The specified white stimulus selected from the one that appeared perfect reflecting diffuser, and normalized by a common factor to derive for Yn that equaled to 100 (16). The Y values of specimens that were measured upon black (Yb) and white (Yw) backgrounds were used to calculate for CR according to equation 4 (11,16): (Fig. 3).

-Opalescence parameter (OP)

**Figure 4:**
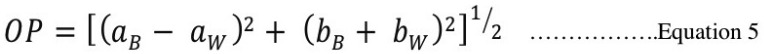
Equation 5.

The values from a* and b* coordinates that were recorded from the specimens placed on a black (B) and a white (W) backgrounds were used to estimate for the opalescence parameter (OP) according to equation 5 (18,29), (Fig. 4).

-Microscopic examination of Y-TZP

The specimens were coated with gold-palladium in sputter coater (K 500X, Emitech, Asford, UK), and further examined with scanning electron microscope (SEM, S-3000N, Hitachi, Tokyo, Japan) and energy dispersive x-ray spectroscopy (Oxford instrument, Oxfordshire, UK) at magnifications of x30,000 magnification.

-Crystalline structure analysis

**Figure 5:**
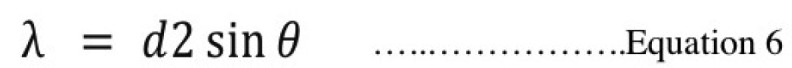
Equation 6.

The crystalline structures of Y-TZP were examined for the relative amount of m- and t- phase using the X-ray diffraction (XRD, PANalytical, Empyrean, Almelo, Netherlands) by scanning with copper k-alpha (Cu Kα) radiation from the Bragg angle (2θ) of 20–40o with 0.02o step size for every 2 seconds’ interval, and compared to the standard database of the joint committee on powder diffraction for calculation d-values using Bragg formula, as Equation 6, (Fig. 5).

Where: λ is X-ray wavelength (0.15418 nm for CuKa), d is normal distance of planes with Miller indices (hkl).

**Figure 6:**
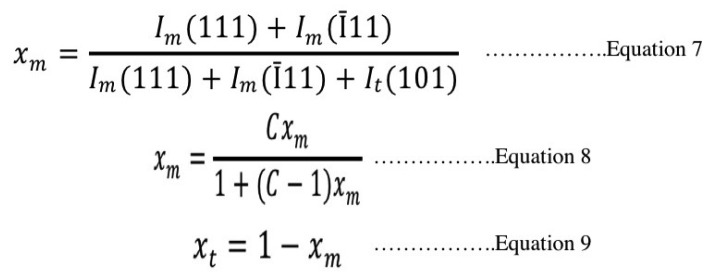
Equations 7,8,9.

The ratio of m- to t- phase was determined by the peaks’ intensities using X’Pert Plus software (Philips, Almelo, Netherland). The mass fraction of m-phase to total phase content was calculated from Garvie-Nicholson formula as shown in Equation 7, and further corrected for non-linearity using Toraya formula as shown in Equations 8 and 9 (30), (Fig. 6).

Where: Im and It: integral intensities of monoclinic and tetragonal phase

C: composition-dependent correction factor (C = 1.32)

Xt and Xm: the Toraya-corrected mass fraction of tetragonal and monolithic zirconia

-Statistical analysis

The data was statistically analyzed using SPSS/PC Version 20 software (IBM, Armonk, NY, USA). An analysis of variance (ANOVA) was used to determine the significant differences in color appearance, translucency, contrast, and opalescent upon different sintering temperatures and sintered-holding times. Post-hoc Tukey’s honest significant difference multiple comparison was determined for the difference between groups at 95% level of confidence.

## Results

**Figure 7:**
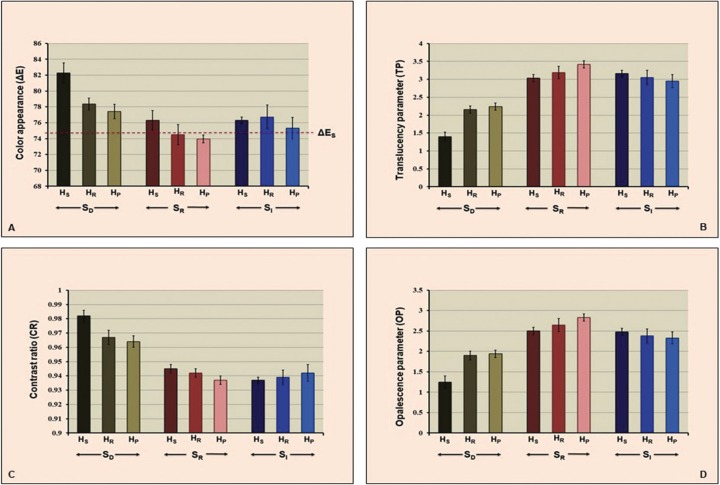
Color appearance (A), translucency parameter (B), contrast ratio (C), and opalescence parameter (D) monolithic zirconia, sintered at decreasing- (SD), regular- (SR), and increasing- (SI) sintering temperature, with shortening- (HS), regular- (HR), and prolonged- (HP) sintered-holding time.

The mean, standard deviation (sd), and 95% confidence interval (CI) of ∆E, TP, CR, and OP for each group were presented in  Table 1 and Figure 7(A-D). The highest mean±sd of ∆E was indicated in the group SDHS (82.28±1.27), followed by SDHR (78.38±0.74), SDHP (77.43±0.91), SIHR (76.73±1.51), SRHS (76.31±1.22), SIHS (76.30±0.43), SIHP (75.32±1.37), SRHR (74.51±1.27) and SRHP (73.97±0.49) as shown in Figure 7(A). The highest mean±sd of TP was indicated in the group SRHP (3.42±0.10), followed by SRHR (3.19±0.17), SIHS (3.16±0.09), SIHR (3.05±0.20), SRHS (3.03±0.10), SIHP (2.95±0.18), SDHP (2.24±0.10), SDHR (2.16±0.10) and SDHS (1.40±0.13) as shown in Figure 7(B). The highest mean±sd of CR value was indicated in the group SDHS (0.982±0.004), followed by SDHR (0.967±0.005), SDHP (0.964±0.004), SRHS (0.945±0.003), SRHR (0.942±0.003), SIHP (0.942±0.006), SIHR (0.939±0.005), SIHS (0.937±0.002) and SRHP (0.937±0.003) as presented in Figure 7(C). The highest mean±sd of OP was indicated in the group SRHP (2.83±0.09), followed by SRHR (2.65±0.16), SRHS (2.50±0.09), SIHS (2.48±0.09), SIHR (2.38±0.17), SIHP (2.33±0.15), SDHP (1.94±0.09), SDHR (1.90±0.11) and SDHS (1.25±0.15) as shown in Figure 7(D).

**Table 1:**
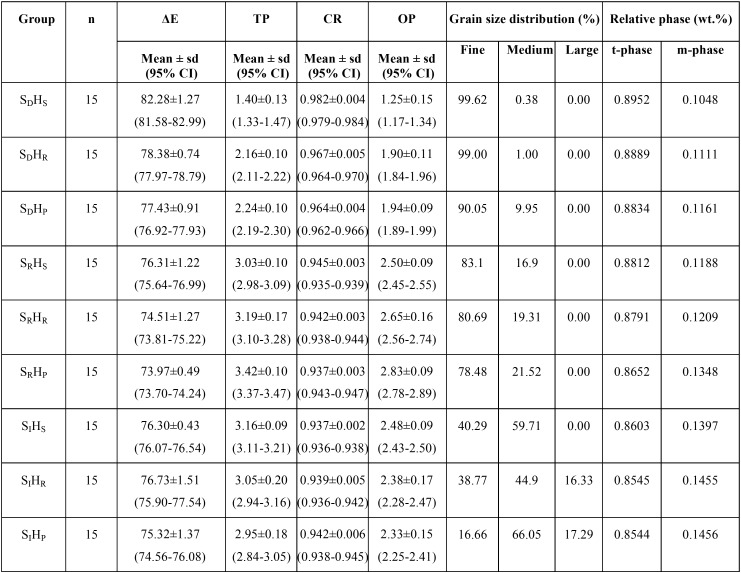
Mean, standard deviation (sd), 95% confidential interval (CI) of color appearance (ΔΕ), translucency parameter (TP), contrast ratio (CR), and opalescent parameter (OP), grain size distribution (%), and relative phase content (wt.%) of monolithic zirconia, sintered at decreasing- (SD), regular- (SR), and increasing- (SI) sintering temperature, with shortening- (HS), regular- (HR), and prolonged- (HP) sintered-holding time.

ANOVA indicated a statistically significant difference in color appearance, translucency, contrast, and opalescence of Y-TZP due to varied sintering temperature and sintered-holding times in sintering process (*p*<0.05), as shown in  Table 2. Post-hoc Tukey’s multiple comparisons indicated that sintering zirconia at a decreasing sintering temperature resulted in significantly higher variation in ∆E and CR than at regular- and increasing-sintering temperature, while sintering zirconia at an increasing sintering temperature resulted in slight variation in ∆E compared to regular sintering temperature (*p*<0.05). However, there was no significant differences in CR between increasing- and regular-sintering temperature (*p*>0.05), as shown in  Table 3. The sintering zirconia at an increasing sintering temperature resulted in comparable TP and OP to regular sintering temperature, while sintering zirconia at a decreasing- resulted in significantly less TP and OP than at increasing- and regular sintering temperature (*p*<0.05), as shown in  Table 3.

**Table 2:**
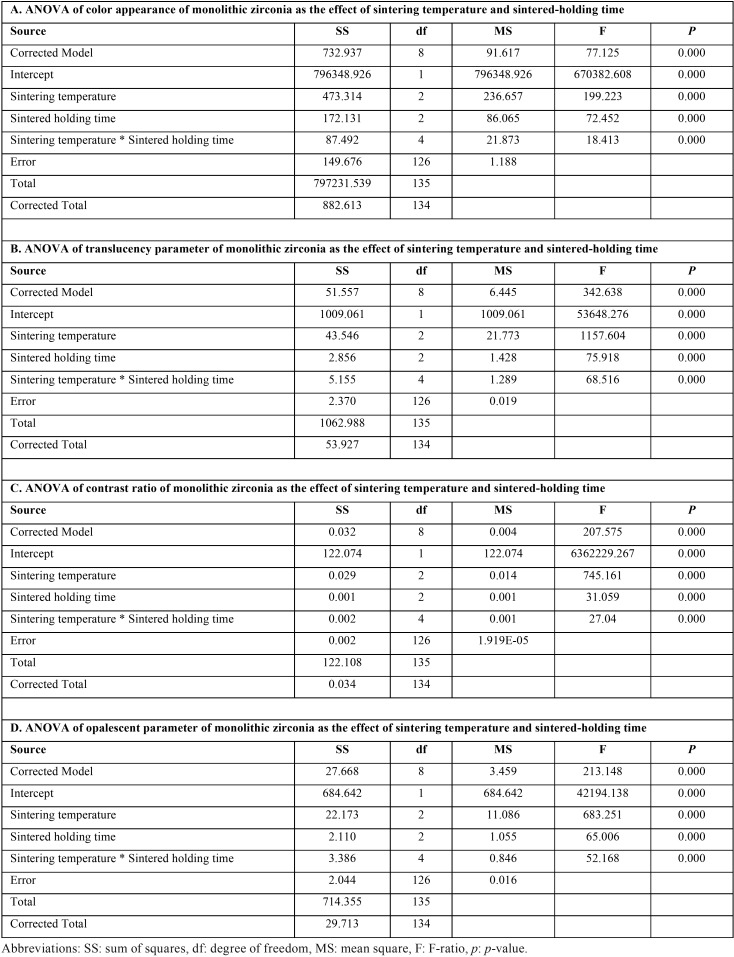
An analysis of variance (ANOVA) of color appearance (A), translucency parameter (B), contrast ratio (C), and opalescent parameter (D) of monolithic zirconia, sintered at decreasing- (SD), regular- (SR), and increasing- (SI) sintering temperature, with shortening- (HS), regular- (HR), and prolonged- (HP) sintering time, indicated the effect of sintering temperature and sintered-holding time.

**Table 3:**
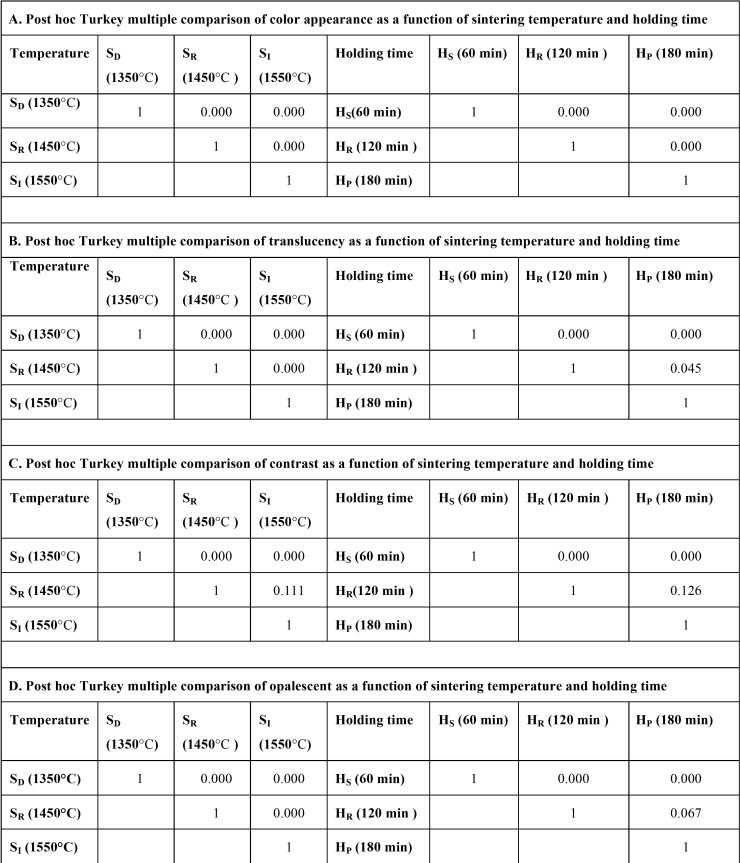
Post hoc Turkey multiple comparisons of color appearance (A), translucency parameter (B), contrast ratio (C), and opalescent parameter (D) of monolithic zirconia, sintered at decreasing- (SD), regular- (SR), and increasing- (SI) sintering temperature, with shortening- (HS), regular- (HR), and prolonged- (HP) sintered-holding time, indicated the effect of sintering temperature and sintered-holding time.

Post-hoc Tukey’s multiple comparisons indicated that shortening sintered-holding time for zirconia resulted in significantly higher variation in ∆E and CR than sintered at regular- and prolonged-sintered holding time, while prolonged sintered-holding time resulted in less variation in ∆E than at regular-sintered holding time (*p*<0.05). However, there was no significant differences in CR between prolonged- and regular-sintered holding time (*p*>0.05), as shown in  Table 3. The prolonged- and regular- sintered-holding time resulted in significantly higher TP, and OP than at shortening sintered-holding time (*p*<0.05). However, there was no significant differences in TP and OP between prolonged- and regular-sintered holding time (*p*>0.05) as shownin  Table 3.

**Figure 8:**
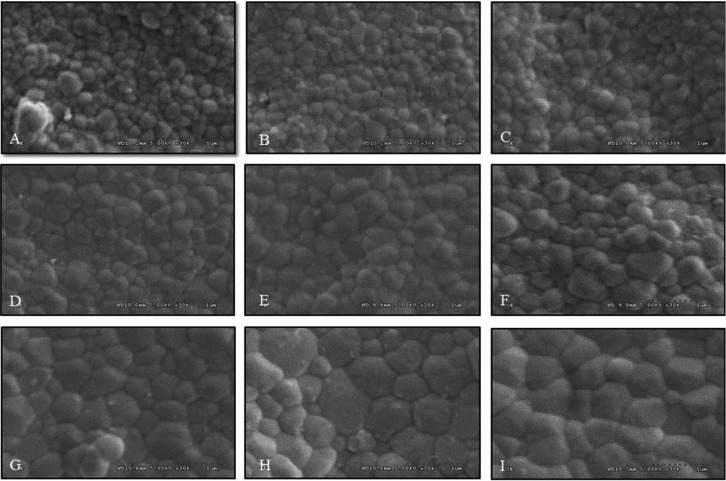
SEM photomicrographs indicated grain size and grain distribution of monolithic zirconia, sintered at decreasing- (A, B, C), regular- (D, E, F), and increasing- (G, H, I) sintering temperature, with shortening- (A, D, G), regular- (B, E, H), and prolonged- (C, F, I) sintered-holding time at X30K magnification.

The SEM photomicrographs were observed for grain size of monolithic Y-TZP. The difference in grain size was illustrated due to vary in sintering process, as shown in  Table 1 and Figure 8. Sintering monolithic Y-TZP at decreasing sintering temperature indicated crystal structures mostly in fine grains (0.1-0.4 μm). Increasing sintering temperature resulted in grain growth and demonstrated an increase in medium- (0.5-0.8 μm) and large-grain size (0.9-1.3 μm). The amount (%) of fine, medium, and large grain sizes were 99.62, 0.38, 0 for SDHS, 99.00, 1.00, 0 for SDHR, 90.05, 9.95, 0 for SDHP, 83.10, 16.9, 0 for SRHS, 80.69, 19.31, 0 for SRHR, 78.48, 21.52, 0 for SRHP, 40.29, 59.71, 0 for SIHS, 38.77, 44.90, 16.33 for SIHR, 16.66, 66.05, 17.29 for SIHP group. The increasing sintering temperature demonstrated the amount of crystal structure in medium grain, more than sintered both in regular- and decreasing- sintering temperatures. It was also demonstrated that longer the holding time, the more grain growth was exhibited as present in Table 1. However, the prolonged holding time seemed to influence less effect on grain growth, when compared to raising sintering temperature. It also indicated defective integration of crystal structure at the grain boundary in the groups that were sintered at decreasing sintering temperature and shortening holding time, while densely compact crystal structures were exhibited upon increasing sintering temperature and prolonged holding time.

The XRD analysis indicated the spectral peaks of samples matched with t- and m- phase of the standard zirconium oxide (ZrO2). The crystalline patterns mostly exhibited in t-phase with minor amount of m-phase in every group. The dominant peak of t-phase was observed at the diffraction angle (2θ degree) of 30.177°, which corresponded to the Miller indices (hkl) crystallographic plane (101). The minor peaks of t-phase were observed at the 2θ degree of 34.607° and 35.172°, which matched to the planes (ī11) and (111) respectively. The m-phases were detected at the 2θ degree of 27.792° and 31.119°, which coincided with the planes (ī11) and (111), respectively. The relative weight percentage (wt.%) t- and m-phase were 0.8544, 0.1456 for SIHP, 0.8545, 0.1455 for SIHR, 0.8603, 0.1393 for SIHS, 0.8652, 0.1348 for SRHP, 0.8791, 0.1209 for SRHR, 0.8812, 0.1188 for SRHS, 0.8834, 0.1161 for SDHP, 0.8889, 0.1111 for SDHR, and 0.8952, 0.1048 for SDHS. The relative amount of m- phase increased as the Y-TZP was sintered either at higher sintering temperature or longer holding time, as presented in Table 1. The phase shifting from t- to m- phase occurred upon increasing sintering temperature and lengthening sintering time. The study indicated that raising sintering temperature and extending sintering time significantly improved color characteristics for better optical appearance, translucency, contrast, and opalescence of nano-sized monolithic Y-TZP.

## Discussion

This study was attempted to evaluate the possibility of achieving better optical appearance of nano-sized monolithic Y-TZP upon altering sintering process. The result signified that varying sintering temperature and sintered-holding time affected to ∆E, TP, CR, and OP of monolithic Y-TZP. This indicated that alteration either sintering temperature or sintered-holding time significantly affected color appearance, translucency, contrast, and opalescence of Y-TZP. Hence, null hypothesis was rejected. The varied sintering parameter is interested in clinical practice because the inquiries always appeared whether the optical characteristics of high translucency monolithic Y-TZP can be achieved to match natural tooth appearance, more translucency, better contrast, and optimized opalescence by altering the sintering temperature and sintered-holding time. It is also doubtful that the possibility of t- to m- phase shifting would occur as altering sintering process might result in alteration in optical characteristics of Y-TZP. The clinical advantage was that an appropriate sintering procedure without jeopardizing color appearance of the zirconia restoration would be beneficial for faster fabricating restoration to be ready for delivery for the patient. This study determined optical characteristics of monolithic Y-TZP by measuring the spectral reflectance, and quantified for ∆E, TP, CR and OP as other studies (11,20,27). Although, the color appearance was significantly changed upon altering sintering process, the amount of changing was within clinically acceptable limit (∆Ediff=3-5) compared to standard color appearance (∆ES) of A2 Vita classical shade guide (Vita Zahnfabrik, Säckingen, Germany), except for the group that was sintered at low sintering temperature and short sintering time (12). This study denoted that sintering monolithic Y-TZP at varied sintering temperature or sintered holding time could apparently detect color difference, but the difference were not be easily perceived by the human eye. Thus based on color perception, the study can assure clinician’s confidence to have the restoration made of monolithic Y-TZP from the dental laboratory without perceivable color appearance difference from the selected standard shade guide even the sintering process was altered.

**Figure 9:**
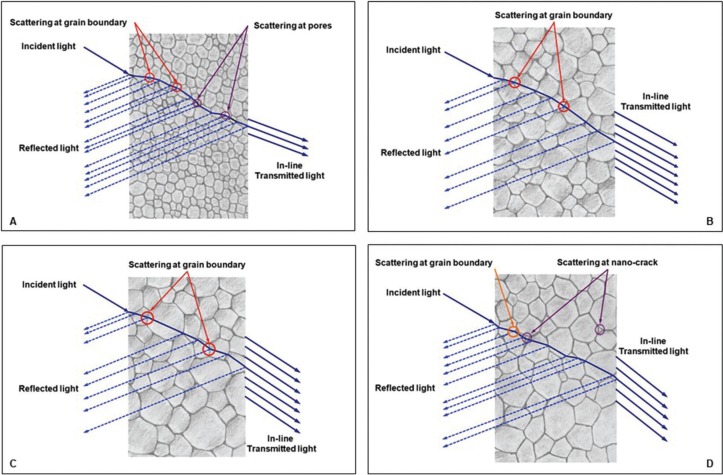
Possible explanation the behavior of light in reflection, scattering, and transmission in relation with grain sized, grain boundary, and pores (A), with increasing translucency upon increasing sintering time (B), increasing sintering temperature (C), while nano-crack in grain boundary exhibited as sintering at extremely high temperature with long duration of sintering time (D).

On the aspect of translucency, it is a prime optical parameter for simulation the natural tooth appearance and is defined as a critical factor in the consideration of material for restorative treatment, especially in the anterior region (8). Translucency relatively associated with the capability of light travelling through the crystal structure, grain, grain boundary, and pore, in which relatively related to their relative refractive index, as shown in Figure 9 (A). The translucency was significantly increasing upon raising sintering temperature and prolong sintered holding time. The translucency of Y-TZP seem to demonstrate minimal difference upon sintering at regular- versus increasing sintering temperature, as well as between regular- and prolong-holding time. On the other hand, the translucency was drastically reduced once the Y-TZP was sintered at decreasing sintering temperature or shortening holding time. This probably related with the maturation of crystalline structures of zirconia as well as the reduction in localized defects on the grain boundaries and the expansion of grain sizes upon raising sintering temperature and time. The increasing of grain size was observed with either raising the sintering temperature (Fig. 9C) or prolonged sintering time (Fig. 9B), which probably capable of diminishing pore and pore distribution at grain boundaries in the poly-crystalline material by facilitating the diffusion capability (Fig. 9B,C). Upon raising the sintering temperature or prolonged holding time, the zirconia particles were capable of joining together, causing reduction in the pore size between the grain boundaries during solid-state diffusion phase, and leading to increasing material density. The result was supported by XRD analysis indicating crystalline phase shifting from t- → m- phase as well as form the SEM photomicrograph indicating grain size enlargement of the nano-sized Y-TZP upon increasing sintering temperature and prolong holding time. As a result of the combination of the porosity reduction and the increasing in density of the sintered nano-sized Y-TZP probably gives rise to the homogeneity of crystalline structure and eventually promotes better specular reflectance, and optical transmission with minimized refraction as manifested in Figure 9(A,B,C). This is probably be a primary reason for this study that indicated the raising sintering temperature to optimal limit can provide better translucency than decreasing sintering temperature as well as the long sintering time tend to achieve higher translucency than either short sintering duration, which was supported by other studies (14,20,24,25,27). However, better increasing translucency of sintered nano-sized Y-TZP slightly deteriorated once the sintering temperature reaching 1550°C, this probably attributed to the extreme increasing in m- phase may accompany by the formation of nano-crack in the grain boundary of m-phase itself that probably act as tiny defect in crystal structure as supported by other studies (11,16). These teeny cracks may initiate further scattering effect and impair diffusion translucency, thus result in interfering favorable translucency as described the Figure 9(D). The formerly reasons also described for CR that decreased as raising sintering temperature and prolong holding time. While opalescent is associated with the amount of oxide such as: ZrO2, Y2O3 that was used for inducing chromatic shade. These oxides always move along the grain boundaries upon raising sintering temperature and prolong holding time, which results in increasing opalescent (26,29). This study indicated a strong correlation among the TP, CR, and OP parameters (r2 = -0.971 for TP versus CR, r2 = 0.991 for TP versus OP, r2 = -0.931 for CR versus OP). This means that as the TP decreases, the OP increases, but the CR decreases, which was in agreement with other studies (16,20,27).

The study suggested that altering sintering process significantly affected optical properties of nano-sized monolithic Y-TZP. It clearly indicated that improving color appearance, translucency, contrast, and opalescent of nano-sized monolithic Y-TZP is possible through raising sintering temperature or prolonged holding time. Vise versa, reducing sintering temperature or shortening holding time can jeopardize color characteristic of nano-sized Y-TZP.

## Conclusions

This study indicated that the optical properties consisting color appearance, translucency, contrast, and opalescence of nano-sized Y-TZP are affected by different sintering process. The significant variation in color appearance was evidenced upon varied sintering temperature and time, but the color appearance still be within clinical acceptable limit of color perception. Nevertheless, significant improvement in translucency, contrast, and opalescent were endorsed through raising sintering temperature and lengthening holding time, which provided much more impact on optical appearance of nano-sized monolithic Y-TZP for serving as aesthetic restoration in clinical practice. Lowering sintering temperature and shortening holding time can compromised optical appearance, even though it’s benefit on restoration fabrication process, in which clinician probably considers for sintering restoration in the area of less visible zone. Ultimately, improvement of optical appearance for nano-sized monolithic Y-TZP is feasible through sintering process by raising sintering temperature or lengthening holding time, which was suggested from this study.

## Clinical significance

Improving optical characteristics of nano-sized Y-TZP are possible through altering sintering process. Sintering nano-sized Y-TZP at high sintering temperature and long sintering time enables achieving better translucency, contrast, and opalescent of restoration and is recommended for sintering process to derive for aesthetic zirconia restoration.
